# Editorial: Plant-derived natural compounds in drug discovery: The prism perspective between plant phylogeny, chemical composition, and medicinal efficacy, volume II

**DOI:** 10.3389/fpls.2023.1324514

**Published:** 2023-11-08

**Authors:** Da-Cheng Hao, Yao-Xuan Wang, Chun-Nian He, Richard W. Spjut

**Affiliations:** ^1^ School of Environment and Chemical Engineering, Biotechnology Institute, Dalian Jiaotong University, Dalian, China; ^2^ Institute of Medicinal Plant Development, Chinese Academy of Medical Sciences and Peking Union Medical College, Beijing, China; ^3^ World Botanical Associates, Bakersfield, CA, United States

**Keywords:** pharmacophylogeny, phylogenomics, chemical constituent, bioactivity, omics

“Pharmacophylogeny” and consequent “Pharmacophylogenomics” aim to disentangle the intricate relationships and connectivity between medicinal plant phylogeny, phytochemical constituents and bioactivities/therapeutic utilities (**Figure 1**) (Hao and Xiao, 2020), so as to promote bioprospecting and benefit plant-based drug R&D. Due to the fact that most medicinal plant researchers are not yet familiar with the theory and methods of pharmacophylogeny, there is still little research on the simultaneous examination of phylogeny/phylogenomics, phytochemistry, and bioactivity. Based on the 13 papers published in the first volume of this Research Topic (Hao et al., 2022a), the volume II has contributed eight brilliant research papers on the metabolomics, network pharmacology and bioactivity of various medicinal plants, covering gymnosperm (Xu et al.), basal angiosperms (Hu et al.), monocot (Hu et al.), basal eudicot (Kakkar et al.), core eudicot (Zheng et al.), Lamiids (Chen et al., Shang et al.) and Campanulids (Zeng et al.), etc. These research findings provide rich compound and pharmacological activity data for further exploration on pharmacophylogeny, facilitating the analysis of distribution patterns of various medicinal compounds and pharmacological activities on the phylogenetic tree (Hao et al., 2022b; Hao et al., 2023), the inference of biosynthetic pathways and therapeutic mechanisms of compounds, and the search for novel drug sources.

**Figure 1:**
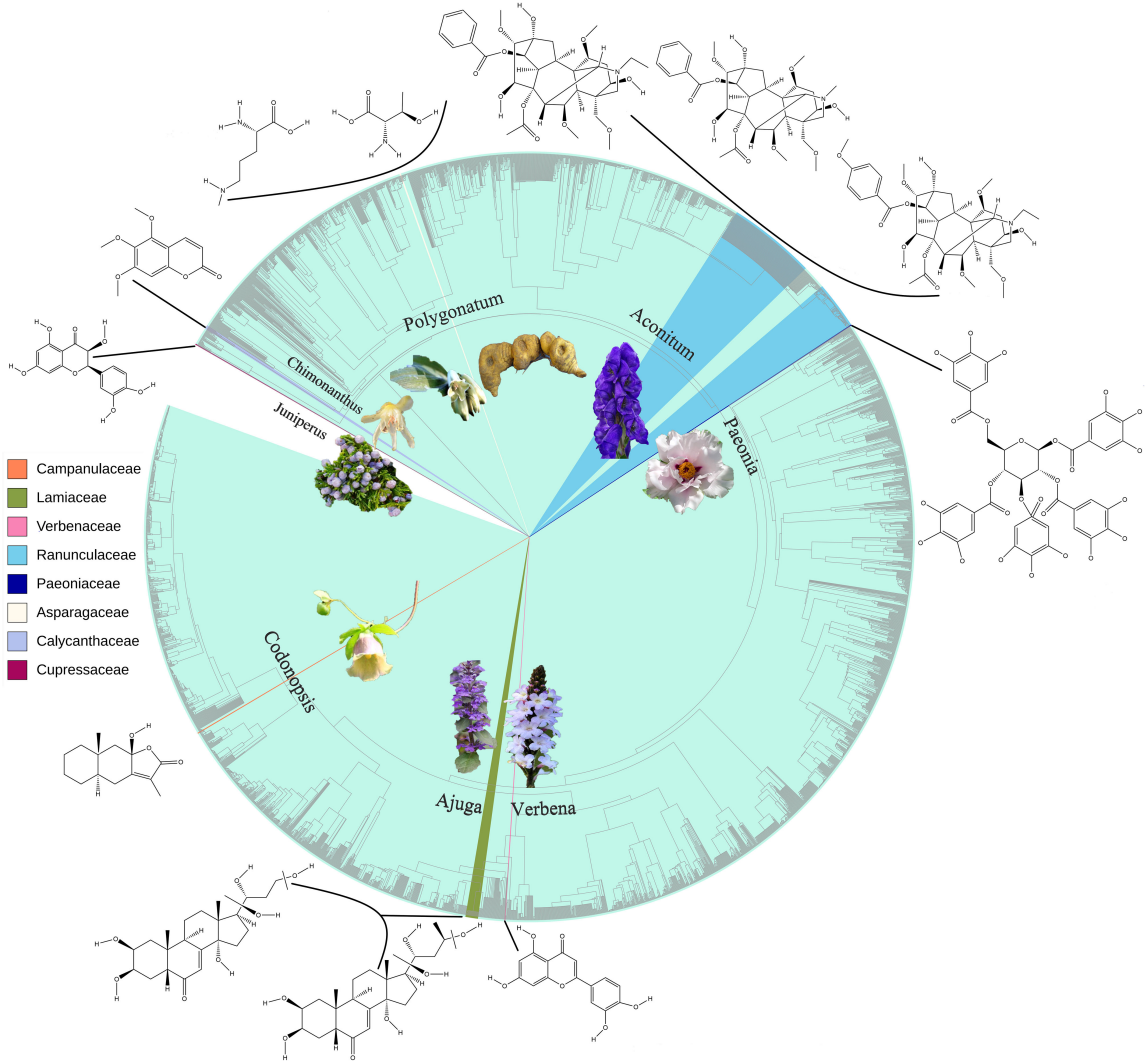
Phylogenetic distribution of taxonomic groups reported in this Research Topic, as well as examples of medicinal compounds therein.

Pharmacophylogeny suggests that curative plants of the related taxonomic groups are more likely to possess the analogous chemical profiles/efficacies, which is supported by the cross-cultural patterns (Lei et al., 2020) and ethnobotany studies (Gaoue et al., 2021). For example, *Aconitum* diterpenoid alkaloids fall into four skeletal types C_18_, C_19_, C_20_, and bisditerpenoid alkaloids (Xiao et al., 2006), and are further divided into 14 sub-groups (Kakkar et al.); many alkaloids of these sub-groups are solely found in different species complex of *Aconitum*. Morphologically, Paeoniaceae was thought to be close to Ranunculaceae (Lu and Tang, 2020), but molecular phylogeny suggested that it is within the order Saxifragales (Chen et al., 2020). Phytochemically, monoterpene glycoside, stilbenes, such as trans-gnetin H and suffruticosol B, and tannins such as 1, 2, 3, 4, 6-penta-O-galloyl-β-D-glucose are predominant in different parts of *Paeonia ostii* (Zheng et al.), while some phenolics and flavonoids such as antioxidants kaempferol and quercitrin are ubiquitously found in numerous taxonomic groups (Zeng et al., Hu et al.). Both Verbenaceae and Lamiaceae belong to the order Lamiales, but the former is closer to Martyniaceae (Chen et al., 2020), while the latter to Mazaceae and Pedaliaceae. Correspondingly, *Verbena officinalis* is salient in its anti-atherosclerotic effect (Chen et al.), possibly due to the actions of flavonoids, steroids, prenol lipids and macrolides, etc., whereas many *Ajuga* species (Lamiaceae) are employed in Traditional Chinese Medicine (TCM) for relieving cough, reducing sputum, and arresting bleeding (Shang et al.).

Currently, pharmacophylogeny is very useful in expanding medicinal plant resources (Hao and Xiao, 2020), authentication/quality control of herbal medicines, predicting the chemicals or bioactive constituents of herbals and identification/quantification of chemicals. Reports on phytometabolites and pharmacological properties of gymnosperms are relatively rare, despite the presence of most types of angiosperm phytometabolites therein. The gymnosperm family Cupressaceae is phylogenetically close to the famous medicinal families Taxaceae and Cephalotaxaceae (Chen et al., 2020; Hao et al., 2021), but its phytochemical profiles and potential therapeutic uses have not been thoroughly characterized. The Cupressaceae genus *Juniperus* is rich in diterpenes, triterpenes, phenolic acids, flavonoids and lignans (Xu et al.), which could be responsible for its anti-inflammatory, antioxidant, antiviral, antibacterial, hypotensive, anticancer, antidiabetic, and neuroprotective properties. *Juniperus* is closer to *Cupressus*, and it is expected that they share some bioactive phytometabolites, which warrants further studies. In the coming years, pharmacophylogeny and pharmacophylogenomics could be more powerful in mining natural products, refining ethnopharmacology understandings, therefore promoting the sustainable conservation and utilization of longstanding/natural pharmaceutical resources.

Against the backdrop of the continuous expansion of anthropogenic activities, the global medicinal plant diversity is facing enormous threats. The goal of volume II is to continue to gain a profound understanding of phylogeny/evolution, phytometabolites and pharmacological effects of selected medicinal genera/families. Although we propose to conduct such explorations in the context of pharmacophylogeny and/or pharmacophylogenomics, it is challenging to recruit such comprehensive manuscripts. This Research Topic presents one endeavor that reconstructed the phylogenetic tree of *Ajuga* based on the whole chloroplast (cp) genome sequences (Shang et al.). The Lamiaceae genus *Ajuga* has around 40-50 species, and 18 are distributed in China (Chen et al., 2020). The cp genome- based phylogeny suggested the closeness of *Ajuga* and the clade consisting of *Amethystea* and *Caryopteris*, and strongly supported a sister relationship between Subsect. Genevense and Subsect. Biflorae, which could be merged into one group. *A. bracteosa* is particularly prevalent and extensively used in folk medicine. Convincingly, it is most closely related to *A. macrosperma*, followed by other taxa of Subsect. Genevense. It is expected that the metabolomic analyses and phytometabolite content determination could reveal the overall similarity of phytometabolite profiles between *A. bracteosa* and phylogenetically related species, and both molecular authentication and chemotaxonomy could be used to differentiate *Ajuga* from common adulterants. The cp genome is an useful genetic resource for phylogeny and evolution studies at both species and subspecies/population levels, while the interspecific chemodiversity could lead to development of novel clinical utility.

The molecular phylogeny and metabolomic information are essential to understand the medicinal value of each genus and to develop alternative medicinal resources. They are also applicable in food medicine continuum (FMC) plants (Hao and Liu, 2023), e.g., *Codonopsis pilosula* (Zeng et al.), *Polygonatum cyrtonema* and *P. sibiricum* (Hu et al.), *Paeonia ostii* (Zheng et al.) and many Lamiaceae species. The active components of TCM and natural medicine are not limited to specialized metabolites (Yang et al., 2022). Many current studies focus on the extraction, separation, activity and quality standards of medicinal components of phytomedicine, while in most cases proteins, carbohydrates and esters were regarded as impurities in pharmaceutical processing, which is influenced by the stereotype of natural medicinal chemistry. With the progress of pharmacophylogeny, phytochemistry and pharmacology, it is clear that some nutrient substances in phytomedicine have certain buff activities, with potential health promoting and disease preventing values. The primary metabolites such as proteins, carbohydrates and nucleotides should be taken into account while studying the chemistry, activity and quality control of phytomedicine. Pharmacophylogeny could help reconsider the contemporary research pathways of TCM and ethnomedicine, and suggest for the development of nutrient substances in FMC plants. Pharmacophylogeny will definitely demonstrate its usefulness in unearthing the cryptic links between phylogenomics and chemotaxonomy of FMC taxa, e.g., those of Liliaceae, Paeoniaceae, Lamiaceae and Campanulaceae, etc.

In summary, according to pharmacophylogeny, taxa in sister phylogeny groups have closely related genetic features (Hao and Xiao, 2020); they are more likely to possess analogous biosynthesis pathways and their chemical repository could be more similar, which is followed by the global resemblance of bioactivity or therapeutic efficacy (Hao et al., 2022b; Hao et al., 2023; Hu et al.). Given that some closely related species may exhibit similar behavioral/defense traits (i.e. strong lineage signals), integrating ecological and evolutionary factors helps to gain a more comprehensive understanding of phytochemical changes in changing environments. Pharmacophylogeny successfully guides the development of novel curative taxa (Hao and Xiao, 2020), while circumventing the limitations of non-holistic approaches and enabling the targeted studies. Hopefully the articles in Volumes I and II of Research Topic can serve as valuable references and enhance researchers’ awareness of pharmacophylogeny, so as to consciously apply relevant methods to the protection, research, and development of medicinal plants.

## Author contributions

D-CH: Conceptualization, Writing – original draft. Y-XW: Formal Analysis, Writing – original draft. C-NH: Writing – review & editing. RS: Writing – review & editing.
